# Recovery from COVID-19 in a Child with Chronic Granulomatous Disease and T Cell Lymphopenia

**DOI:** 10.1007/s10875-020-00896-2

**Published:** 2020-10-27

**Authors:** Vasudha Mantravadi, Suong T. Nguyen, S. Celeste Morley, Jeffrey J. Bednarski, Maleewan Kitcharoensakkul, Megan A. Cooper

**Affiliations:** 1 Department of Pediatrics, Division of Allergy and Pulmonary Medicine, Washington University School of Medicine, St. Louis, MO 63110 USA; 2 Department of Pediatrics, Division of Infectious Diseases, Washington University School of Medicine, St. Louis, MO 63110 USA; 3 Department of Pediatrics, Division of Hematology/Oncology, Washington University School of Medicine, St. Louis, MO 63110 USA; 4 Department of Pediatrics, Division of Rheumatology/Immunology, Washington University School of Medicine, 660 S. Euclid Ave, St. Louis, MO 63110 USA

**Keywords:** Chronic granulomatous disease, COVID-19, immunodeficiency

To the Editor:

Severe acute respiratory syndrome coronavirus 2 (SARS-CoV-2), first discovered in December 2019, has led to an ongoing, unprecedented global pandemic due to coronavirus disease 2019 (COVID-19). This novel single-stranded RNA virus infects airway epithelial and other cell types expressing surface angiotensin-converting enzyme 2, with clinical manifestations ranging from asymptomatic infection to acute respiratory distress syndrome and severe organ dysfunction [1, 2]. Symptom onset is typically between 4 to 12 days of incubation. Older age and co-morbidities such as cancer, diabetes, and cardiovascular disease increase the risk for more severe disease, and a hyper-inflammatory response to the virus plays a key role in the multi-organ damage that is seen in severe cases [3]. Case reports of patients with inborn errors of immunity (IEI) suggest that the presence of B cells may contribute to immunopathology compared to a milder course in patients lacking B cells [4, 5]. A larger international retrospective cohort reported the clinical course of 94 patients with IEI, primarily adults, with approximately 30% of patients having asymptomatic or mild infection, and major risk factors for disease severity including co-morbidities also present in the general population, with a fatality rate of ~ 10% [6]. This cohort included 4 patients with chronic granulomatous disease (CGD), 3 of whom did well, while one patient with concomitant *Burkholderia* infection died. Interrogation of the immune response in patients with severe COVID-19 has also revealed the importance of the type I interferon (IFN) pathway in controlling infection, including loss-of-function variants in genes in this pathway and neutralizing antibodies to type I IFNs [7, 8]. Here, we present the case of COVID-19 in a patient with CGD and T cell deficiency due to failed hematopoietic cell transplantation (HCT) who recovered with supportive care.

**Fig. 1:**
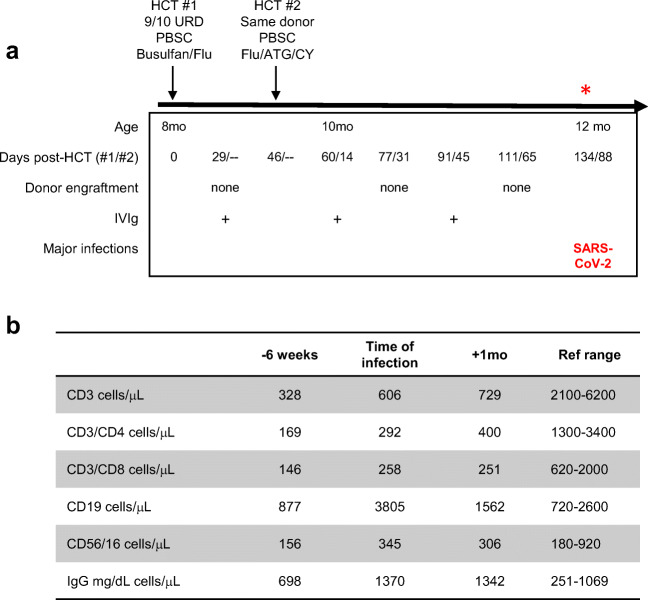
Timeline of SARS-CoV-2 infection post-HCT and immune phenotype. **a** The patient received 2 HCTs from the same donor without evidence of donor chimerism in the myeloid or T cell compartments. He received 3 doses of intravenous immunoglobulin (IVIg). **b** Lymphocyte counts and IgG level before and after infection with SARS-CoV-2. References ranges for age shown. ATG, anti-thymocyte globulin; CY, cyclophosphamide; Flu, fludarabine; HCT, hematopoietic cell transplantation; PBSC, peripheral blood stem cells

The patient is a 12-month-old African-American boy with X-linked CGD due to a missense variant in *CYBB*, who had undergone allogeneic hematopoietic stem cell transplant twice using the same donor without engraftment. Unfortunately, there was no evidence of engraftment in the myeloid or T cell compartment following either HCT (Fig. 1a). He was negative for EBV and CMV and did not have any known viral infections post-transplant. Neutrophil oxidative burst remained undetectable, and his T cell numbers  remained low due to HCT conditioning (Fig. 1b). He was receiving prophylactic trimethoprim-sulfamethoxazole and itraconazole. Intermittent doses of IgG replacement therapy (400 mg/kg dosing) were administered post-transplant, the most recent ~6 weeks prior to presentation (Fig. 1a). He presented with 2 days of nasal congestion 12 days after exposure to a family member with COVID-19. Nasopharyngeal PCR testing was positive for SARS-CoV-2. He was afebrile without respiratory distress and was well hydrated. Laboratory studies were notable for transaminitis with alanine transaminase of 726 units/L and aspartate transaminase of 219 units/L, increased from his chronic mild transaminitis related to itraconazole therapy. He had T cell lymphopenia, elevated B cell numbers, elevated IgG (Fig. 1b), and a normal neutrophil count. Inflammatory markers were increased including erythrocyte sedimentation rate (ESR) at 29 mm/h (normal range 3–13 mm/h) and C-reactive protein (CRP) of 28.2 mg/L (normal less than 10 mg/L). He was discharged home to continue supportive care.

He presented to the emergency department 7 days later with fever, labored breathing, a cough, and diarrhea. Additional family members were also positive for SARS-CoV-2 at this point. On physical examination, the patient was afebrile, tachycardic (178 beats/min), and tachypneic (54 breaths/min), but normotensive and saturating 99% on room air. He had mild subcostal retractions but no adventitious breath sounds or other abnormal findings. Chest radiograph revealed airspace opacities in the right middle and upper lung and left lung base. Laboratory evaluation showed hyponatremia to 126 mmol/L, improved transaminitis with alanine transaminase of 148 units/L and aspartate transaminase of 60 units/L, an increased white blood cell count (16.4 K cells/μL) with neutrophilic predominance (absolute neutrophil count of 11.4 K cells/μL), and further elevation of inflammatory markers, ESR 38 mm/h and CRP 155.7 mg/L. Given his tachycardia, markers to assess for possible myocardial injury from COVID-19 were checked and were normal, including undetectable troponin I, lactate dehydrogenase of 334 units/L, and total creatine kinase of 65 units/L. Electrocardiogram showed sinus tachycardia and possible right ventricular hypertrophy. A hypercoagulable state was evidenced by his elevated fibrinogen level of 556 mg/dL (normal range 170–400 mg/dL), D-dimer of 5232 ng/mL (normal less than 499 ng/mL), and thromboelastography showing shortened reaction and clot formation times. Prothrombin time and partial thromboplastin time were normal. He was started on empiric cefepime for potential secondary bacterial pneumonia given his underlying CGD. His prophylactic antibiotics were continued.

The patient was admitted to the pediatric intensive care unit for close monitoring, but never required any respiratory support, and was quickly transferred to the general pediatric floor the following day. His hyponatremia had resolved with intravenous hydration. He exhibited mild respiratory distress when febrile but remained on room air throughout the hospitalization and improved with supportive measures, including antipyretic administration. He did not receive any antiviral therapy for SARS-CoV-2. After bacterial blood cultures remained negative for 48 hours, cefepime was discontinued. With improvement of liver enzymes, his prophylactic itraconazole was increased to 5 mg/kg twice daily to achieve therapeutic levels. He was discharged after 3 days and was afebrile. His cough continued to improve at home by the following week. Repeat testing for SARS-CoV-2 by PCR remained positive 11 and 25 days following discharge; however, he remained asymptomatic. He did not have detectable SARS-CoV-2 IgG antibodies 2 months following discharge but had a negative viral PCR from a nasopharyngeal swab.

The majority of pediatric COVID-19 cases are mild, and while our patient was hospitalized, he recovered without respiratory support, antiviral therapy for SARS-CoV-2 infection, or anti-inflammatory therapy for COVID-19. Several aspects of his disease course are consistent with the current literature on COVID-19: the time course of initial symptom onset 10 days after infectious exposure, followed by development of pulmonary infiltrate within 8–9 days after symptom onset [1]; the presence of transaminitis, which was attributed to itraconazole, given his prior history of demonstrating the same side effect, but may have also be an indicator of immune mediated or direct viral cytopathic injury to the liver; the presence of hypercoagulability, which can reflect a hyper-inflammatory state triggered by the infection; and finally, the increase in neutrophil-to-lymphocyte ratio that is often seen in COVID-19 and correlates with disease severity [2, 3].

Neutrophils employ several anti-pathogen defense mechanisms, including NADPH-mediated reactive oxygen species production and neutrophil extracellular trap formation, which have been implicated in lung tissue injury and hyper-inflammation seen in severe COVID-19 [3]. Based on this, one might speculate that impaired neutrophil activity in CGD might protect against the exaggerated inflammatory response and consequent tissue damage observed in severe COVID-19, consistent with the mild disease course observed in our patient. On the other hand, T cell lymphopenia might be expected to predispose to an inability to control or restrict SARS-CoV2 infection. Our patient had T cell lymphopenia secondary to his failed bone marrow transplant. Although he had a mild disease course, consistent with minimal inflammation, he also exhibited prolonged viral shedding, as detectable by nasopharyngeal sampling for 25 days after presentation. Delayed viral clearance would be consistent with his T cell deficiency. There are limited studies of COVID-19 in primary immunodeficiency patients, but the effect of immunodeficiencies on COVID-19 risk and how they may lead to enhanced susceptibility or even protection will improve as our understanding of the immunopathogenesis of this infection evolves and clinical cases are described.
